# High-Throughput 3-D Monitoring of Agricultural-Tree Plantations with Unmanned Aerial Vehicle (UAV) Technology

**DOI:** 10.1371/journal.pone.0130479

**Published:** 2015-06-24

**Authors:** Jorge Torres-Sánchez, Francisca López-Granados, Nicolás Serrano, Octavio Arquero, José M. Peña

**Affiliations:** 1 Department of Crop Protection, Institute for Sustainable Agriculture (IAS-CSIC), Cordoba, Spain; 2 Institute of Agricultural Research and Training (IFAPA-Alameda del Obispo), Cordoba, Spain; University of Calgary, CANADA

## Abstract

The geometric features of agricultural trees such as canopy area, tree height and crown volume provide useful information about plantation status and crop production. However, these variables are mostly estimated after a time-consuming and hard field work and applying equations that treat the trees as geometric solids, which produce inconsistent results. As an alternative, this work presents an innovative procedure for computing the 3-dimensional geometric features of individual trees and tree-rows by applying two consecutive phases: 1) generation of Digital Surface Models with Unmanned Aerial Vehicle (UAV) technology and 2) use of object-based image analysis techniques. Our UAV-based procedure produced successful results both in single-tree and in tree-row plantations, reporting up to 97% accuracy on area quantification and minimal deviations compared to in-field estimations of tree heights and crown volumes. The maps generated could be used to understand the linkages between tree grown and field-related factors or to optimize crop management operations in the context of precision agriculture with relevant agro-environmental implications.

## Introduction

The geometric measurements of the agricultural trees, such as tree height and crown volume, serve to monitor crop status and dynamic, to analyse tree production capacity and to optimise a number of agronomic tasks, such as water use, nutrient application, pruning operations and pest management. Conventionally, the main tree dimensions are measured by hand after an intensive field work and next the crown volume is estimated with equations that treat the trees as regular polygons or by applying empiric models [1]. However, collecting this data at the field scale is very time-consuming and generally produces uncertain results because of the lack of fit of the real tree to the geometric models or to the great variability in orchards that can affect the suitability of models based on in-field measurements. Among the technological alternatives, the Light Detection And Ranging (LiDAR) laser scanners and the stereo vision systems by using terrestrial or remote-sensed measurements are currently the most relevant [2]. However, these techniques have also their own limitations in real tree orchards. On the one hand, although the terrestrial devices are very precise to measure tree architecture [3–5], they are inefficient in large spatial extents and are difficult to use in hard-to-reach field areas. On the other hand, remote-sensed data collected with piloted aircrafts and satellites do not often fulfil the technical requirements (e.g., sufficient spatial resolution or number of stereoscopic pairs) needed to detect the 3-dimensional (3-D) characteristics of agricultural trees in most cases [2].

In recent years, a new aerial platform has joined the traditional ones: the Unmanned Aerial Vehicles (UAV) or drones [6,7]. Several investigations [8] have demonstrated the advantages of the UAVs in comparison to airborne or satellite missions regarding its low cost and greater flexibility in flight scheduling [9], which make UAV technology a proper tool for farmers and researchers to monitor crops at the field scale [10]. In addition, the UAV can automatically flight at low altitudes and with large overlaps, which permit the acquisition of ultra-high spatial resolution images (in the range of a very few centimetres) and the generation of the Digital Surface Model (DSM) using automatic photo-reconstruction methods that are based on the “Structure from Motion” approach for 3-D reconstruction. As a consequence, recent investigations have focused on the generation of DSM with UAVs [11] and its interpretation over agricultural areas [12–14].

However, in order to take full advantage of this technology, another primary step involves the implementation of robust and automatic image analysis procedures capable of retrieving useful information from the images. To reach a high level of automation and adaptability, we propose the application of object-based image analysis (OBIA) techniques. OBIA overcomes some limitations of pixel-based methods by grouping adjacent pixels with homogenous spectral values after a segmentation process and by using the created “objects” as the basic elements of analysis [15]. Next, OBIA combines spectral, topological, and contextual information of these objects to address complicated classification issues. This technique has been successfully applied in UAV images both in agriculture [16,17], grassland [18] and urban [19] scenarios.

In this article, we report an innovative procedure for a high-throughput and detailed 3-D monitoring of agricultural tree plantations by combining UAV technology and advanced OBIA methodology. After the DSM generation with UAV images, this procedure automatically classifies every tree in the field and computes its position, canopy projected area, tree height and crown volume. For training and testing purposes, we used olive plantations as model systems and selected several sites with a variable degree of tree shapes and dimensions, both in conventional single-tree and in row-structured plantation systems. Efficacy of the procedure was assessed by comparing UAV-based measurements and in-field estimations. In addition, effects of spectral and spatial resolutions on the entire process were evaluated in each type of plantation by performing different flight missions in which two flight altitudes and two sensors (a conventional low-cost visible-light camera and a 6-band multispectral color-infrared camera) were separately tested. Finally, time required by each stage of the full process was weighted according to the flight mission performed.

## Materials and Methods

**Fig 1:**
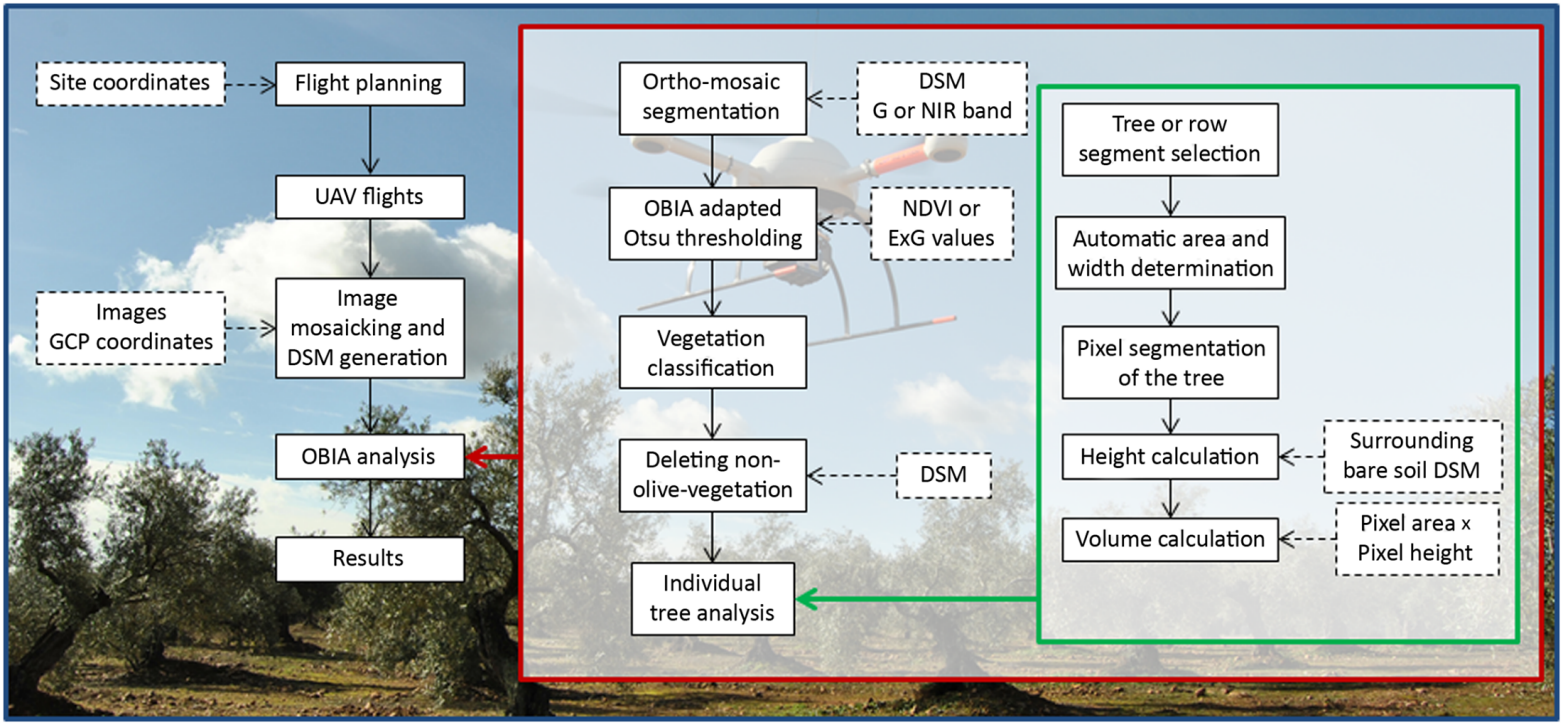
Flowchart of the entire procedure for 3-D monitoring of agricultural tree plantations by combining UAV technology and object-based image analysis. (Abbreviations: 3-D (three dimensional); GPS (Global Position System); UAV (Unmanned Aerial Vehicle); GCP (Ground Control Point); DSM (Digital Surface Model); G (Green band); NIR (Near Infra-Red band); OBIA (Object-Based Image Analysis).

The full procedure consisted on three main phases (Fig 1): 1) the acquisition of very high spatial resolution remote images with an unmanned aerial platform, 2) the generation of orthomosaics and DSMs by applying close-range photogrammetry methods, and 3) the application of advanced object-based algorithms to analyse the images and to retrieve the position and the geometric features of each tree or tree-row in the whole field. Next, each stage is described in detail.

### Description of the UAV and the sensors

**Fig 2:**
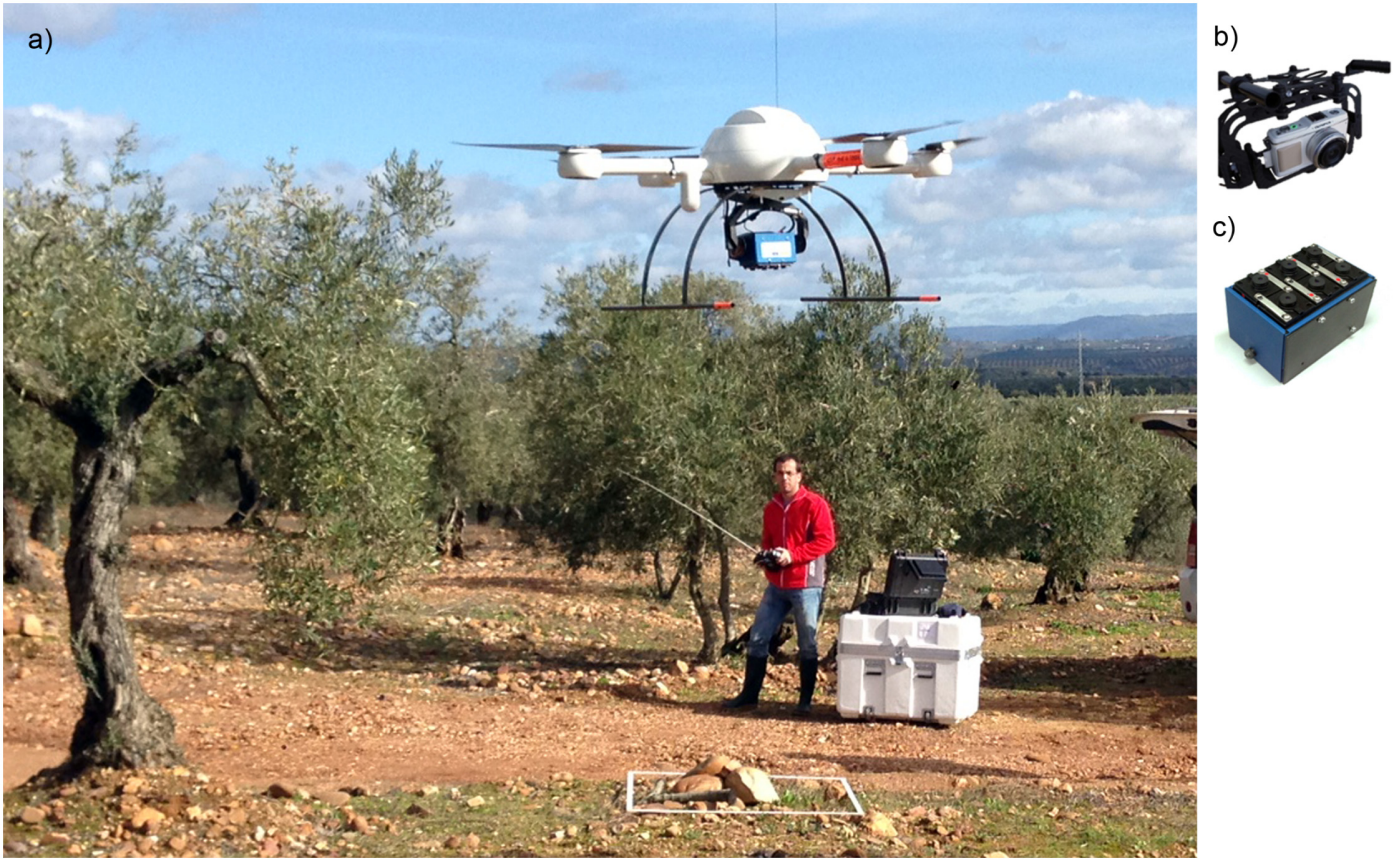
The quadrocopter UAV, model md4-1000, taking off in one of the studied fields (a) and the sensors used in this investigation: the visible-light camera (b) and the multispectral camera (c). The individual in this figure has given written informed consent to publish his photograph.

The UAV used in this investigation was a quadrocopter with vertical take-off and landing (VTOL), model MD4-1000 (microdrones GmbH, Siegen, Germany) (Fig 2a). This UAV is equipped with four brushless motors powered by a battery and it can be manually operated by radio control (1000 m control range) or it can fly autonomously with the aid of its Global Position System (GPS) receiver and its waypoint navigation system. The VTOL system makes the UAV independent on a runway, which allows the use of the UAV in a wide range of different situations, e.g., even on steep olive orchards.

Two sensors were separately tested: 1) a still point-and-shoot visible-light camera, model Olympus PEN E-PM1 (Olympus Corporation, Tokyo, Japan) (Fig 2b), and 2) a six-band multispectral camera, model Tetracam mini-MCA-6 (Tetracam Inc., Chatsworth, CA, USA) (Fig 2c). On the one hand, the visible-light camera produces 12.2 megapixel format (4,032 x 3,024 pixels) images in true colour (Red, R; Green, G; and Blue, B, bands) with 8-bit radiometric resolution, which are stored in a secure digital SD-card. It is equipped with a 14–42 mm zoom lens, although it was fixed at 14 mm focal length for these works. The camera’s sensor size is 17.3 x 13.0 mm and the pixel size is 0.0043 mm. These parameters are needed to calculate the image resolution on the ground or, i.e., the ground sample distance (GSD) as affected by the flight altitude (Eq 1). On the other hand, the multispectral camera is a lightweight (700 g) sensor composed of six individual digital channels arranged in a 2x3 array. Its sensor size is 6.66 x 5.32 mm and the pixel size is 0.0052 mm. Each channel has a focal length of 9.6 mm and a 1.3 megapixel (1,280 x 1,024 pixels) CMOS sensor that stores the images on a compact flash CF-card. The images were acquired with 8-bit radiometric resolution. The camera has user configurable band pass filters (Andover Corporation, Salem, NH, USA) of 10-nm full-width at half-maximum and centre wavelengths at B (450 nm), G (530 nm), R (670 and 700 nm), R edge (740 nm) and near-infrared (NIR, 780 nm). More details about the sensors and UAV configuration can be consulted in [20].

$$\text{GSD}=\;\frac{\text{Sensor Pixel Size }\times \text{Flight Altitude}}{\text{Focal Length}}$$ (1)

### Study sites and UAV flight missions

**Fig 3:**
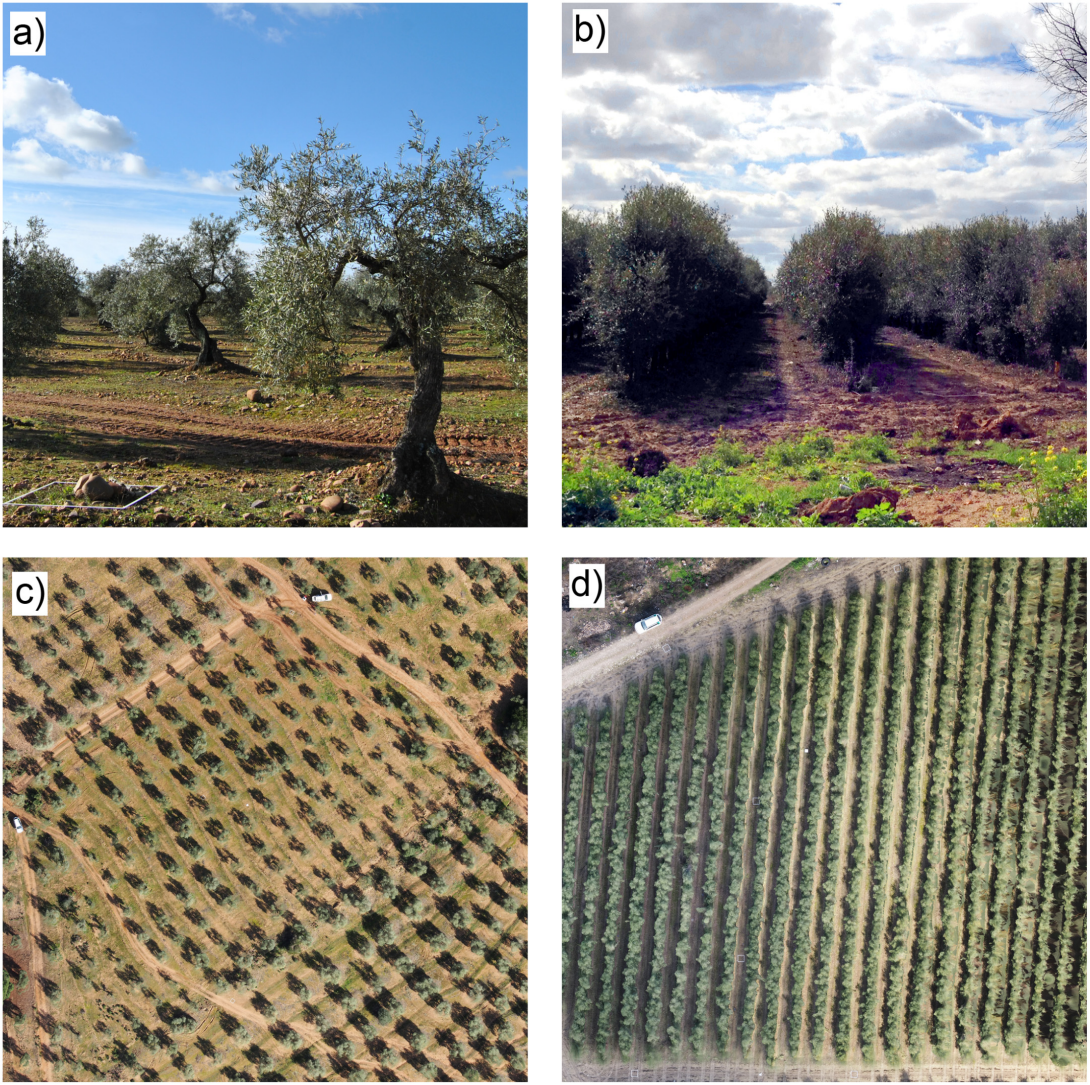
On-ground (top) and aerial (down) views of two plantations studied in this investigation with single-tree (a, c) and tree-row (b, d) patterns, respectively.

We used olive plantations as model systems to develop and evaluate our procedure and selected four different sites with a variable degree of tree shapes and dimensions, as well as with two different plantation patterns: two fields with a traditional single-tree distribution (Fig 3a and 3c) and two fields with the trees in rows (Fig 3b and 3d). The fields were identified by four different letters to facilitate the reading of the article, as follows: field A: located in the public research farm “Alameda del Obispo” in Cordoba, field B: a private farm located in Adamuz (Cordoba province), field C: a private farm located in Pedro Abad (Cordoba province), and field D: a private farm located in Villacarrillo (Jaen province).

Different flight missions with the two sensors mounted independently in the UAV were performed in every field (Table 1). In the private farms, the flights were authorized by a written agreement between the farm owners and our research group. On the one hand, the UAV route was configured with the visible-light camera to continuously take photos at an interval of 1 second, which resulted to a forward lap of 90% at least. In this case, the UAV flied in line with a side lap of 60%. With the multispectral camera, the UAV route was programmed to stop in every acquisition point due to camera technical limitations for continuum shooting (slower processing speed). In this case, the images were taken with a side lap and a forward lap of 60%. In all flight missions, the image overlapping was high enough to apply the 3-D reconstruction procedure in the next stage. According to these flight configurations, the visible-light camera can cover roughly 10 ha and 20 ha and the multispectral camera roughly 3 ha and 6 ha, at 50 and 100 m altitude, respectively, in each regular 30-minutes flight.

**Table 1:** Description of the tree plantations and of the flight operations performed in each field.

Tree plantation			Flight operation^2^		
Field ID	Location^1^	Plantation pattern (tree spacing)	Flight date	Sensor	Flight altitude (m)
A	Cordoba (37.855N, 4.806W)	Single-trees (7x7 m)	21^st^ Aug, 2013	Visible-light	50, 100
				Multispectral	50
B	Adamuz (37.992N, 4.505W)	Single-trees (8x8 m)	21^st^ Feb, 2014	Visible-light	50,100
				Multispectral	50,100
C	Pedro Abad (37.960N, 4.466W)	Tree-rows (3.75x1.3 m)	21^st^ Feb, 2014	Visible-light	50,100
				Multispectral	50,100
D	Villacarrillo (38.113N, 3.163W)	Tree-rows (8x4 m)	12^th^ May, 2014	Visible-light	50,100
				Multispectral	100

^1^ Lat/Lon coordinate system; Datum WGS84.

^2^ Multispectral images of the field “B” at 100 m altitude and of the field “D” at 50 m altitude were not taken due to technical problems.

The acquired images had different spatial resolutions according to the technical characteristics of the sensor and to the flight altitude as follows (Eq 1): 1) the visible camera flying at 50- and 100-m altitudes produced RGB images with a GSD of 1.53 and 3.06 cm, respectively; and 2) the multispectral camera flying at 50- and 100-m altitudes produced multispectral images with a GSD of 2.71 and 5.42 cm, respectively. These experiments aimed to assess the influence of spatial and spectral resolution on the accuracy of the DSM generation and on the performance of the OBIA tasks (see sections 2.3 and 2.4, respectively). The flight routes fulfilled the requirements that were established by the Spanish National Agency of Aerial Security for maximum flight altitude allowed for UAVs, which is currently fixed at 120 m [21].

### Generation of the ortho-mosaics and of the Digital Surface Models (DSM)

Mosaicking and DSM generation were performed using the Agisoft PhotoScan Professional Edition software (Agisoft LLC, St. Petersburg, Russia). The mosaicking process was fully automatic with the exception of the manual localisation of a few ground control points that were taken in each field. The entire automatic process involves three principal stages: 1) aligning images, 2) building field geometry, and 3) ortho-photo generation. First, the camera position for each image and the common points in the images were located and matched, which refined the camera calibration parameters. Next, the DSM was built based on the estimated camera positions and the images themselves. This second stage needs high computational resources and it usually takes a long time in the case of using many high-resolution images. Finally, the separated images were projected over the DSM, and the ortho-mosaic was generated. The DSM is a 3-dimensional polygon mesh that represents the overflown area and reflects the irregular geometry of the ground and the tree crowns. The DSMs were joined to the ortho-mosaics as Tiff files, which produced a 4-band multi-layer file from the visible-light camera (RGB bands and the DSM) and a 7-band multi-layer file from the multispectral sensor (6 bands and the DSM). A more detailed explanation of the PhotoScan functioning is given in [22].

### Object-based image analysis (OBIA) procedure

**Fig 4:**
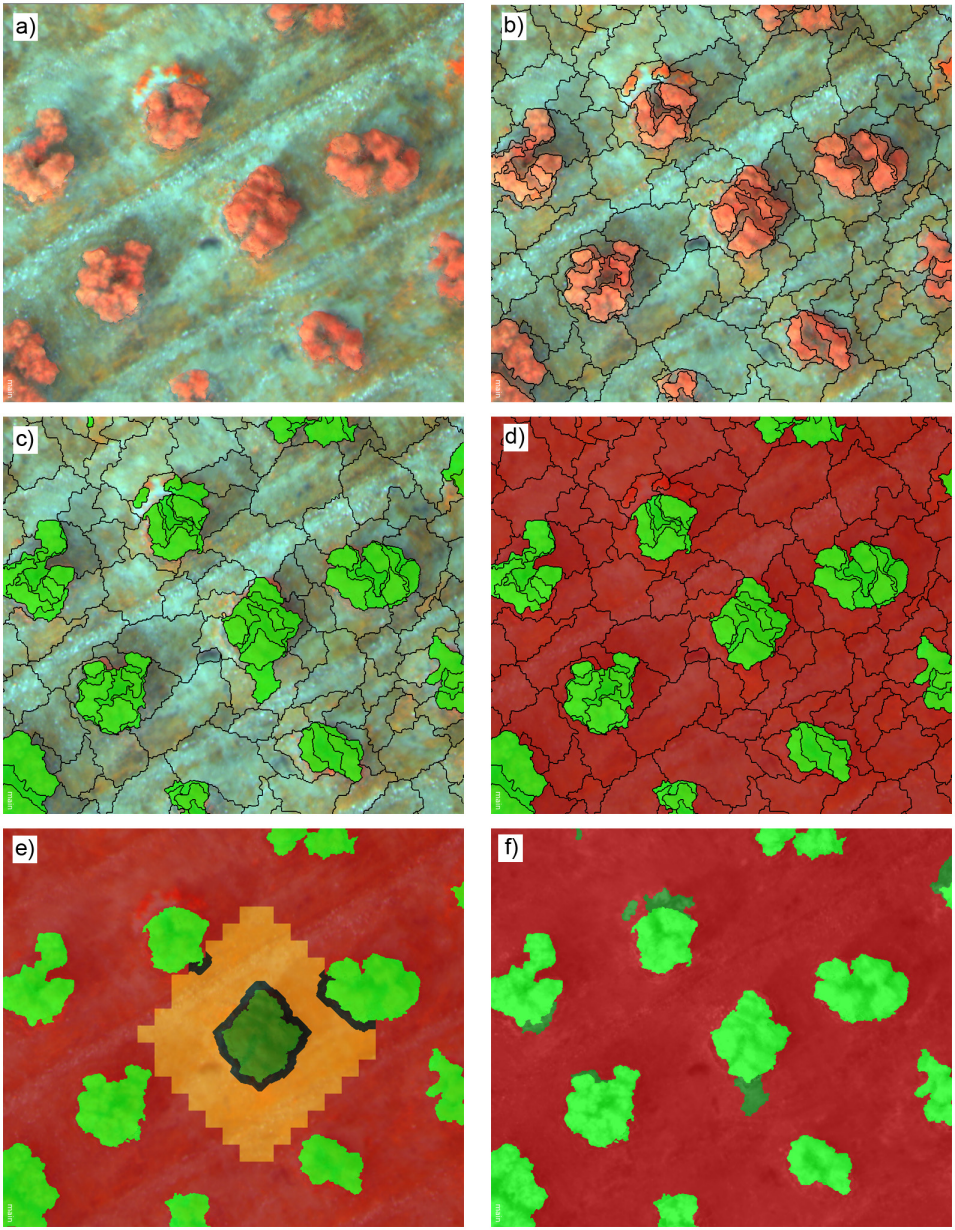
Partial views of each phase of the OBIA procedure developed to classify agricultural-tree plantations: a) Mosaicked image composed of the spectral information (in this example, multispectral bands) and the DSM data, b) segmentation output, c) classification of the vegetation objects (in green color), d) removal of the herbaceous vegetation, e) identification of the bare-soil area (in orange color), which is used as the base line to calculate the height of the neighbouring vegetation objects (in dark green color), and f) classification of the trees (in bright green color), herbaceous vegetation (in dark green color) and bare soil (in red color) based on the spectral information and the vegetation height.

The multi-layer files that were generated in the previous stage were analysed with an original OBIA algorithm that was developed with the eCognition Developer 9 software (Trimble GeoSpatial, Munich, Germany). This algorithm is auto-adaptive to any remote image with independence of the plantation pattern and it can be apply with minimum user interference. The algorithm is composed of a number of rules that are grouped in four consecutive main phases (Fig 4):

1. Image segmentation: The image was segmented into objects using the multiresolution segmentation algorithm [23] (Fig 4a). For a better delineation of the trees, the layers in which the trees were more prominent, i.e., the DSM layer and either the Green band from the visible-light images or the NIR band from the multispectral image, were weighted to 1, and the remaining layers were weighted to 0. The scale parameter varied in the function of the sensor and the spatial resolution, and the remaining segmentation parameters were 0.6, 0.4, 0.5 and 0.05 for colour, shape, smoothness and compactness, respectively (Fig 4b).
2. Image classification: The classification task took advantage of the capacity of certain vegetation indices to enhance the discrimination of vegetation targets. In this investigation, the Excess Green index (ExG, Eq 2, [24]) for the visible-light images and the Normalised Difference Vegetation Index (NDVI, Eq 3, [25]) for the multispectral images were calculated. Then, a threshold for vegetation discrimination was established using Otsu’s automatic thresholding method [26] as adapted to the OBIA framework [27]. After the application of the threshold to the vegetation indices values, vegetation was isolated from bare soil (Fig 4c). Next, the herbaceous vegetation surrounding the trees was isolated considering the DSM layer and applying the criterion of vegetation with low height compared to surrounding soil (Fig 4d). The vegetation pixel height was derived from the relative difference of the DSM values between the pixels of each individual vegetation object and the pixels of the bare soil surrounding each object. In this step, only the bare soil pixels that were very close to each vegetation object were specifically selected as the baseline for height calculation, eliminating potential errors due to the terrain slope (Fig 4e).
  $$\text{ExG}=\text{2g}-\text{r}-\text{b; being g=}\frac{\text{G}}{\left(\text{R+G+B}\right)}\text{; r=}\frac{\text{R}}{\left(\text{R+G+B}\right)}\text{; b=}\frac{\text{B}}{\left(\text{R+G+B}\right)}$$ (2)
  $$\text{NDVI}=\frac{\left(\text{NIR}-\text{R}\right)}{\left(\text{NIR}+\text{R}\right)}$$ (3)
3. Computing and mapping of the 3-D features (canopy width, length and projected area, tree height and crown volume) of each individual tree or tree-row: The vegetation objects that were classified as trees in the previous stage were merged to compound each individual tree or tree-row. This merging operation was performed in a new level created over the original segmentation. Therefore, a hierarchical segmentation structure was generated, in which the merged objects (trees or tree-rows) were in the upper level and the segmented objects were in the bottom level. At this point, the geometric features such as width, length and projected area of the tree canopy and the tree height were automatically calculated by applying a looping process in which each tree or tree-row was individually identified and analysed. Next, the crown volume was calculated by integrating the volume of all of the individual pixels (bottom level) that were positioned below each tree or tree-rwo (upper level) in the hierarchical structure. In this operation, the height and area of every tree pixel were multiplied to obtain the pixel volume, and the tree volume was subsequently derived by adding the volume of all of the pixels below each olive tree or tree-row. This step was performed at the pixel level, which permitted dealing with the irregular shape of every tree or tree-row and consequently avoiding the errors that are usually produced in empirical estimations due to inexact comparisons of the trees or tree-rows to regular solids.
4. Delivery of the map outputs: After computing the tree geometrical features, the OBIA procedure automatically exported such information as vector (e.g., shapefile format) and table (e.g., excel or ASCII format) files for further analysis and applications.

### Training and validation of the methodology

This investigation was conducted following a training/validation procedure. The training stage was performed in the field A and the field C as representative of single-tree and tree-row plantations, respectively, and consisted of testing the flight configuration and image acquisition. This stage also involved visual tests of image quality and evaluation of the aptitude of the mosaicked images and their associated DSMs to build the tree structures and to retrieve their geometric features. In addition, we also developed the OBIA algorithms in the training fields. Next, the validation procedure was performed in the field B and the field D as representative of single-tree and tree-row plantations, respectively. Three geometric features, namely the projected area of the canopy, tree height and crown volume, were evaluated by comparing the UAV-estimated values and the on-ground values observed in the validation fields.

In the case of the projected area, the observed values were derived by manually delineating the shape of all of the trees or tree-rows over the mosaicked images that were generated in each flight route. Then, the classification outputs that were generated by the OBIA algorithms were overlapped with the manual classifications to compute the area of coincidence for each tree or tree-row and to calculate the overall classification accuracy in each scenario (Eq 4).

$$\text{Overall Classification Accuracy (\%)}=\text{100}\times \left(\frac{\text{Area correctly classified}}{\text{Total area}}\right)$$ (4)

In the case of height and volume quantification, 24 trees in the field B and 30 trees in the field D were selected for validation. All of the trees were georeferenced with a GPS device to locate their position in the mosaicked images. In addition, the tree height and canopy diameter were manually measured with a ruler, and the crown volume was estimated assuming an ellipsoid form and applying a validated method (Eq 5) for olive tree geometric measurements [28]. However, the crown volumes were not calculated in the field D because its row structure impeded the identification of tree edges in the longitudinal axis.

$${\text{Crown volume (m}}^{\text{3}}\text{)}=\frac{\pi }{\text{6}}\times {\left(\frac{\text{(Canopy length axis)}+(\text{Canopy width axis)}}{\text{2}}\right)}^{\text{2}}\times \left(\text{Tree height}\right)$$ (5)

The efficacy of the entire procedure (mosaicked images, DSM layer and OBIA algorithms) to measure the tree height and crown volume of individual trees was assessed by comparing the UAV-estimated values and on-ground values that were observed in the 54 validation trees. Then, the overall accuracy and its associated average error (Eq 6) that were attained in each scenario (two validation fields and several flight altitudes), as well as the root mean square error (RMSE) and correlation coefficient that were derived from the regression fit, were calculated to quantify the influence of each factor on monitoring each studied tree variable.

$$\text{Average Feature Error}=\frac{\sum_{i=0}^{n}\left|\text{(UAV}-{\text{measured Feature}}_{\text{i}})-(\text{Field}-{\text{observed Feature}}_{\text{i}})\right|}{\text{Number of trees}}$$ (6)

## Results and Discussion

### Quality of ortho-mosaic and DSM generation

**Fig 5:**
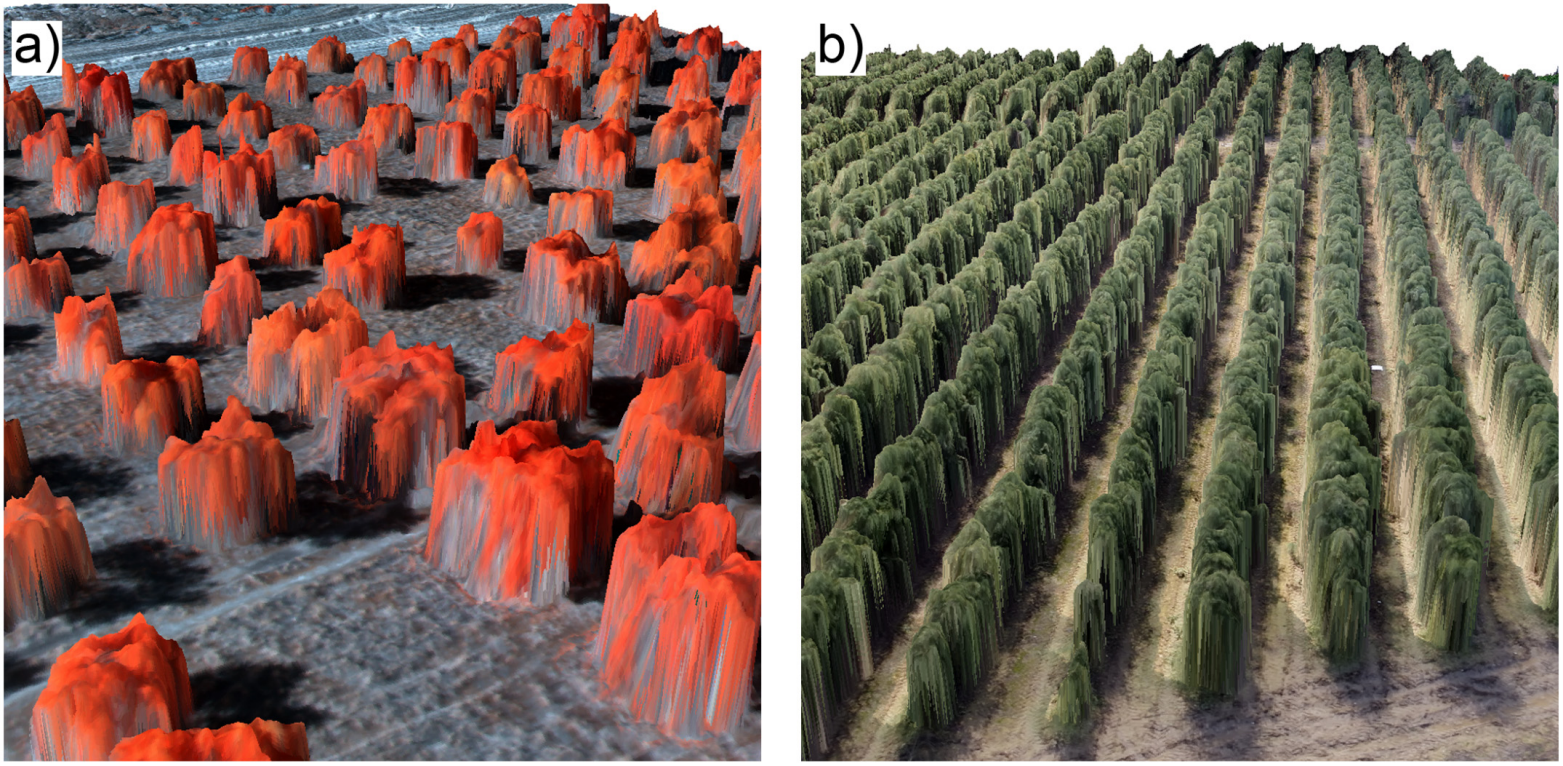
3-D representation of a single-tree plantation generated with a multispectral sensor (a) and of a tree-row plantation generated with a visible-light camera (b).

The Fig 5a and 5b show the 3-D representation generated in two fields with single-tree and tree-row systems, respectively. Each image was composed of two products: the ortho-mosaic and its associated DSM. Both plantations were modelled in 3-D with high accuracy, showing the irregular shape of the trees and of the tree-rows including typical crown gaps and branch distribution, which allowed computing tree volume regarding the real crown shape. The ortho-mosaics were successfully created in all the studied scenarios (four fields, two sensors and two flight altitudes), with the exception of the images that were collected with the multispectral sensor over the tree-row plantations. However, the quality of the DSMs was variable as affected by the sensor type and the tree plantation system (Table 2). With the independence of the flight altitude, the DSMs were satisfactorily generated in both single-tree plantations (field A and field B) with the multispectral sensor and in both tree-row plantations (field C and field D) with the visible-light camera. In fact, more than 96% of the trees in the single-tree fields and the 100% of the rows in the tree-row fields were correctly modelled, and only some mixing effects were observed after the image analysis process in the DSMs that were generated with the visible-light images that were captured at a 100-m altitude. In contrast, the DSM generation procedure partially failed with the visible-light images collected in the single-tree fields (mainly in the field B). In these cases, the 3-D structure of some of the trees was not built and, consequently, the mosaicked images showed some blurry areas. On the one hand, we observed that the procedure for 3-D reconstruction with the visible-light images was more problematic in the trees with a low canopy density. As a consequence, we hypothesized that the low colour contrast between some trees and their surrounding bare soil area was the reason of the errors in the generation of the DSM in the separated-tree cropping system scenarios. In fact, greater errors were obtained in the field B, where the colour of many trees was similar to that of bare soil, than in the field A, where a greater contrast between the trees and the bare soil was observed. On the other hand, the multispectral sensor totally failed in both row-tree plantations due to certain difficulties of the 3-D reconstruction software to find common points during the image alignment process. We attributed these errors to insufficient spatial resolution of this sensor in order to match similar points in overlapped images taken over homogeneous targets, as we also observed in additional investigations on herbaceous crops.

**Table 2:** Number and percentage of trees or tree-rows correctly reconstructed during the DSM generation procedure as affected by the sensor type and the flight altitude in each of the studied fields.

				Trees or tree-rows correctly reconstructed	
Field ID	Plantation pattern	Sensor	Fligh altitude (m)	Number	%
A	Single-trees	Visible-light	50	65	73
			100	86	97
		Multispectral	50	89	100
B	Single-trees	Visible-light	50	27	20
			100	74	55
		Multispectral	50	135	100
			100	130	96
C	Tree-rows	Visible-light	50	9	100
			100	9	100
		Multispectral	50	0	0
			100	0	0
D	Tree-rows	Visible-light	50	10	100
			100	10	100
		Multispectral	50	10	100

### Classification accuracy as affected by the flight altitude

**Fig 6:**
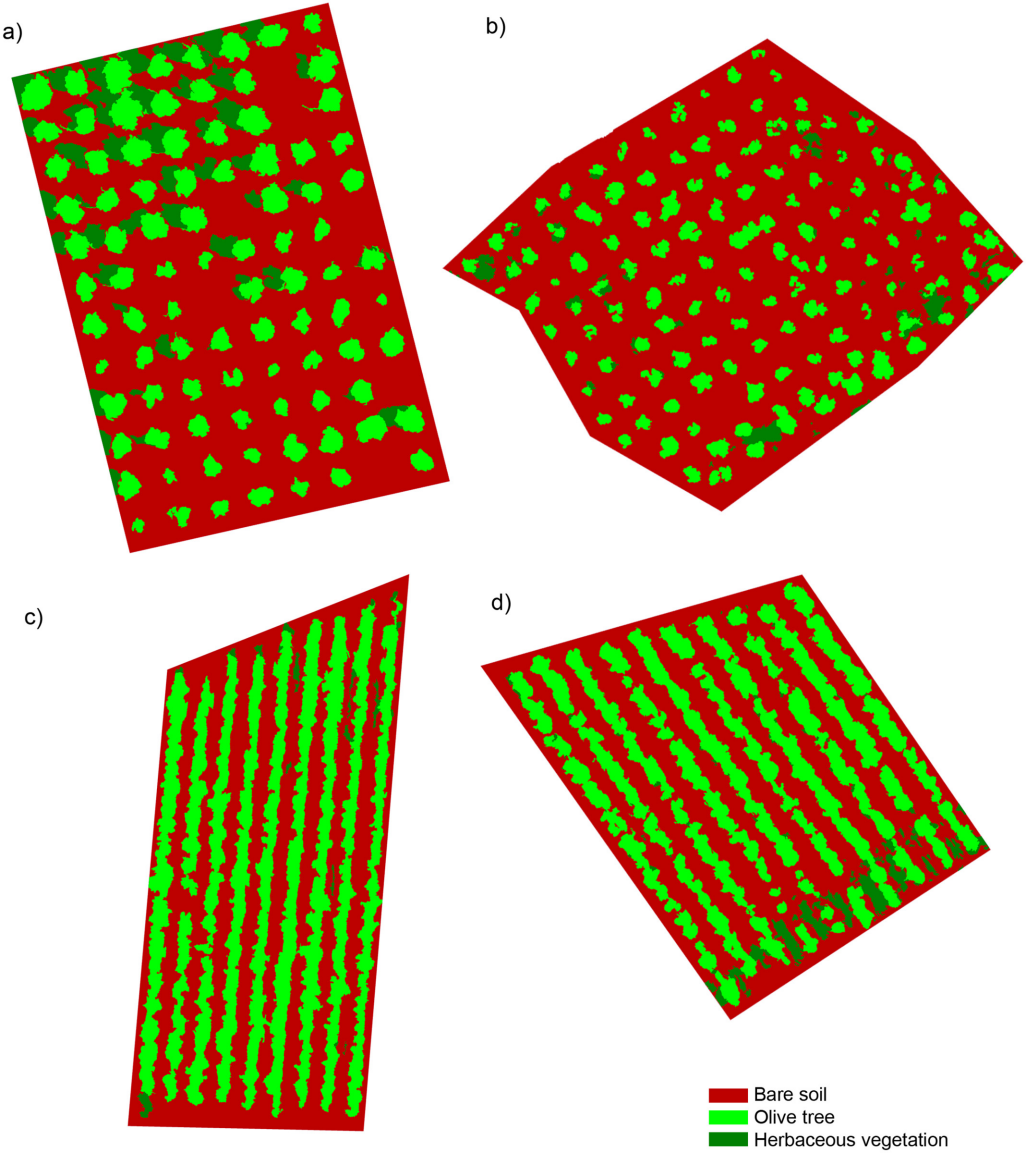
Classification outputs generated by the OBIA algorithm developed in this investigation. Our innovative algorithm automatically classified individual trees (a, b) or tree-rows (c, d), herbaceous vegetation and bare soil areas and, simultaneously, computed the geometric features (projected canopy area, tree height and crown volume) of each individual tree or tree-row in the whole plantation.

After building the 3-D models of the four studied fields, we applied our original OBIA procedure in order to classify the remote images (Fig 6) and to measure the geometric features of each individual tree or tree-row, whichever applies. Our OBIA procedure was designed to auto-adapt, with minimum user intervention, to any agricultural tree plantation with a similar crop patterns (e.g., citrus groves, vineyards or *Prunus* orchards). The algorithms were submitted to a training/validation procedure, in which the images collected in the fields A and C were used for creating and training the OBIA algorithm and the images collected in the fields B and D were used to validate the results (section 2.5). The classification procedure achieved an overall accuracy of approximately 95% or even higher in the most cases (Table 3). With the independence of the sensor used and the field studied, minor differences in the classification accuracy were observed for different flight altitudes. The visible-light and the multispectral sensors captured images with pixel sizes ranging from 1.5 cm to 3.1 cm and from 2.7 cm to 5.4 cm at the studied flight altitudes, respectively. The high spatial resolution imagery that was generated by both sensors, even at a 100-m flight altitude, permitted the correct identification of the tree canopy, which produced a successful classification in every case. Generally, at least four pixels are required to detect the smallest objects within an image [29]. Accordingly, the sensors that were used in this investigation were adequate for analysing individual tree or tree-row structures with a minimum dimension of approximately 10x10 cm or even smaller if the flight altitude was lower than 100 m. Therefore, these results recommend collecting the UAV remote images at the highest altitude allowed by the aviation regulations (in Spain, 120 m maximum [21]) in order to capture the maximum ground area in each image and to consequently optimise the flight mission length and image ortho-mosaicking process.

**Table 3:** Overall accuracy attained by the OBIA algorithm in the classification stage.

Field ID	Plantation pattern	Sensor	Flight Altitude (m)	Overall Accuracy (%)
A	Single-trees	Multispectral	50	97.4
B	Single-trees	Multispectral	50	96.9
			100	94.5
C	Tree-rows	Visible-light	50	93.1
			100	86.8
D	Tree-rows	Visible-light	50	96.4
			100	95.7

### Quantification of the tree geometric features (canopy area, tree height and crown volume)

#### Canopy area

**Fig 7:**
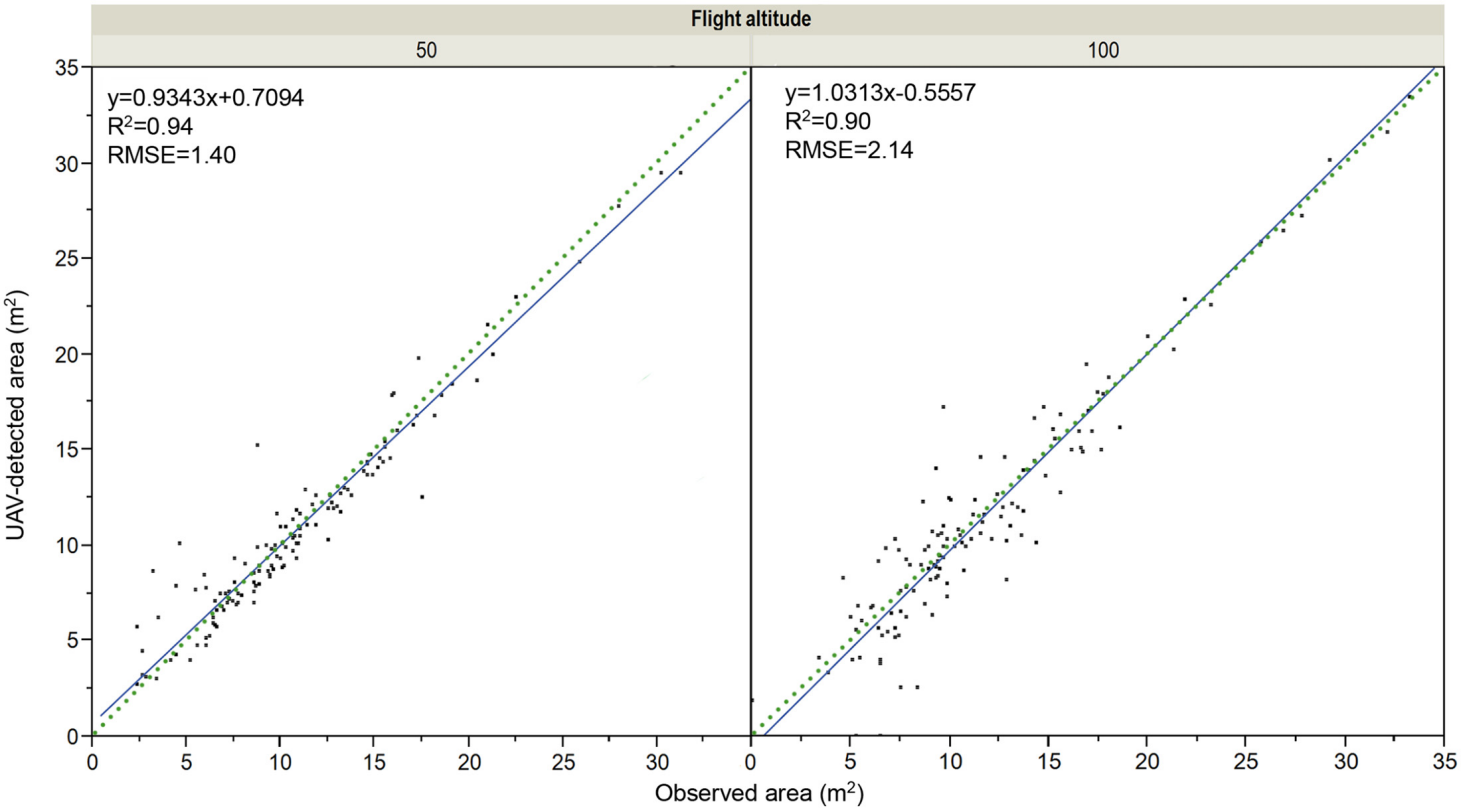
Classified vs. observed tree projected area after applying the OBIA algorithm in the remote images collected at 50 m (left) and 100 m (right) of flight altitude over the field B. The solid line is the fitted linear function and the dotted line is the 1:1 line.

The relation between canopy projected area classified by the OBIA procedure and the observed values at the 50-m-altitude images was close to the 1:1 line (R^**2**^ = 0.94, RMSE = 1.44 m^**2**^), although it tended to a subtle underestimation of the trees or groups of nearby trees larger than 20 m^**2**^ (Fig 7). With the 100-m-altitude images, this relationship was also close to the 1:1 line, but the correlation coefficient (R^**2**^ = 0.90) and the RMSE (2.14 m^**2**^) was slightly worse than at the ones reported at 50-m-altitude. The canopy areas of all the trees were estimated with minimum errors in the images at both flight altitudes, which demonstrated algorithm robustness. In fact, the tree canopy edges were automatically defined with high precision even in zones with surrounding herbaceous vegetation, where discriminating vegetation types is a complicate task due to their similar spectral responses. In this case, tree classification was facilitated by incorporating the DSM information (i.e., pixel height) as an input layer in the segmentation procedure and, afterwards, by using an automatic height-based thresholding method for identifying the tree canopy edges.

#### Tree height

**Fig 8:**
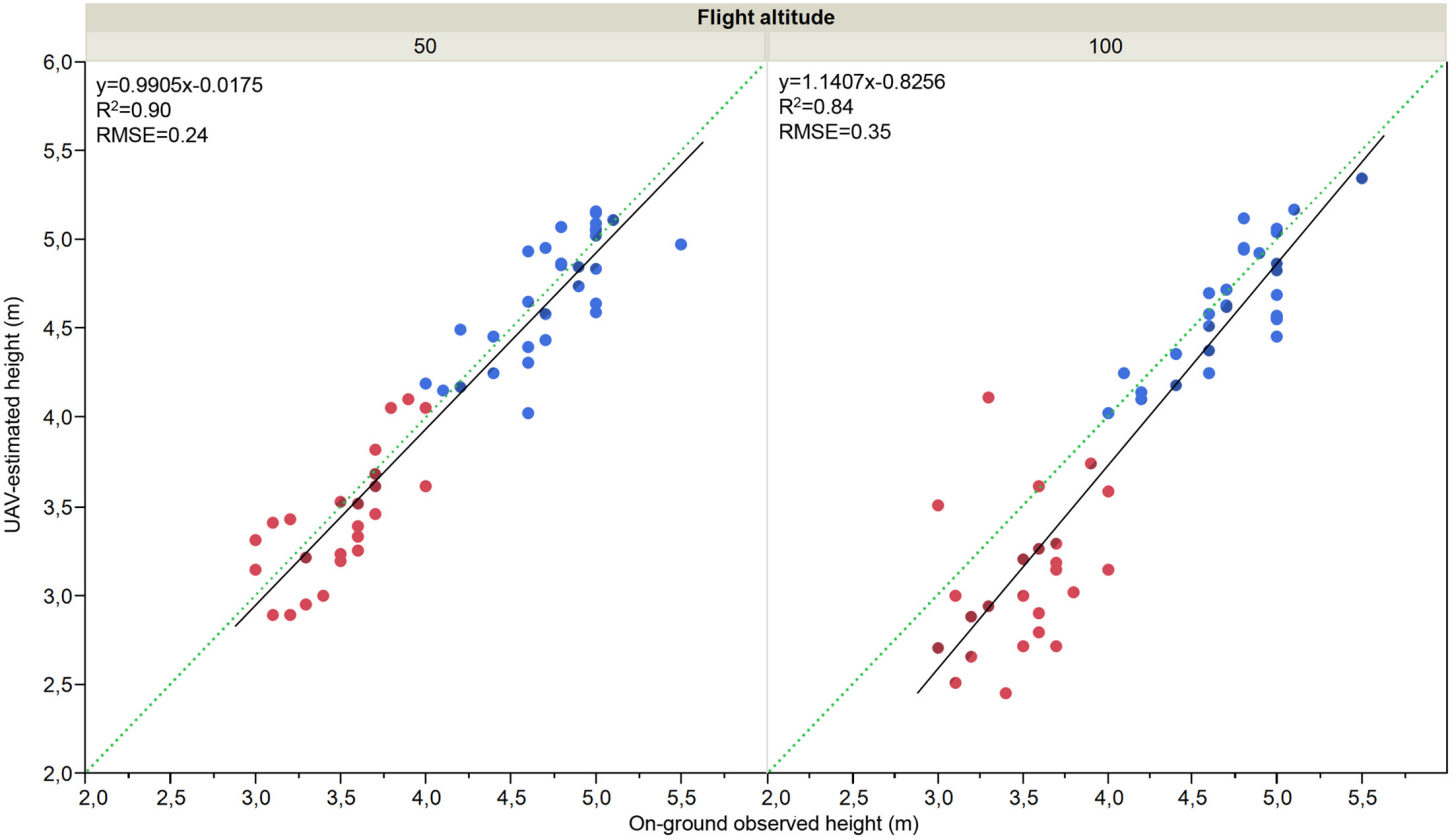
Comparison between on-ground observed and UAV-estimated tree height values measured from the images captured at 50 m (left) and 100 m (right) of flight altitude, respectively. The results in the tree-row plantations (blue dots) were obtained with the visible-light camera and in the single-tree plantations (red dots) with the multispectral sensor. The solid line is the fitted linear function and the dotted line is the 1:1 line.

Tree height was estimated with unprecedented accuracy, reporting averaged errors in the range of 0.17–0.18 m from the images captured with the visible-light camera and of 0.22–0.53 m from the images captured with the multi-spectral camera (Table 4). Previous investigations with a similar image-based UAV technology reported RMSE values on tree height estimations in the range of 0.33–0.39 m [12] and of 0.44–0.59 m [30] in olive-tree and palm-tree plantations, respectively. An essential difference with these investigations refers to the image analysis technique used to compute the tree parameters in each case. We implemented an OBIA algorithm instead of the pixel-based filtering algorithms applied in [12,30]. OBIA has various advantages for analysing high-resolution images where the pixels can be aggregated to create new elements (e.g., trees) with an evident spatial pattern. Here, the OBIA algorithm identified all the trees in the plantation with very high accuracy (Table 3) and treated each of the trees as an individual object. This tree-by-tree procedure can exactly select the local maxima (in the tree apex) and minima (in the surrounding on-ground base-line) extreme pixels that are used by the OBIA algorithm to calculate the height of each individual tree. By comparing the on-ground observed and the UAV-measured height values, the coefficient of determination was 0.90 and 0.84 for the UAV-images captured at 50-m and 100-m flight altitudes, respectively (Fig 8). The regression line was very close to the 1:1 line with the results derived from the images captured at 50-m flight altitude, although some under-estimation was obtained from the 100-m-altitude images, particularly in the case of trees shorter than 4 m height. In general, the UAV-based estimations of the tree heights only deviated a few centimetres from the on-ground measurements. However, these deviations were greater in the shortest trees and using the highest flight altitude, which likely denotes a positive relationship between both variables. For this application, these errors are tolerable but, if required, vertical estimations could be improved by reducing the flight altitude according to tree heights, although further investigation is needed to determine the optimal flight configuration for image-based 3-D photo-reconstruction.

**Table 4:** Tree height quantification errors (average and standard deviation) accounted in the validation fields.

				Tree height quantification error	
Field ID	Plantation pattern	Sensor	Flight Altitude (m)	Averaged	Standard deviation
B	Single-trees	Multispectral	50	0.22 m (6.32%)	3.41
			100	0.53 m (15.55%)	8.12
D	Tree-rows	Visible-light	50	0.18 m (3.75%)	3.06
			100	0.17 m (3.54%)	3.16

#### Crown volume

**Fig 9:**
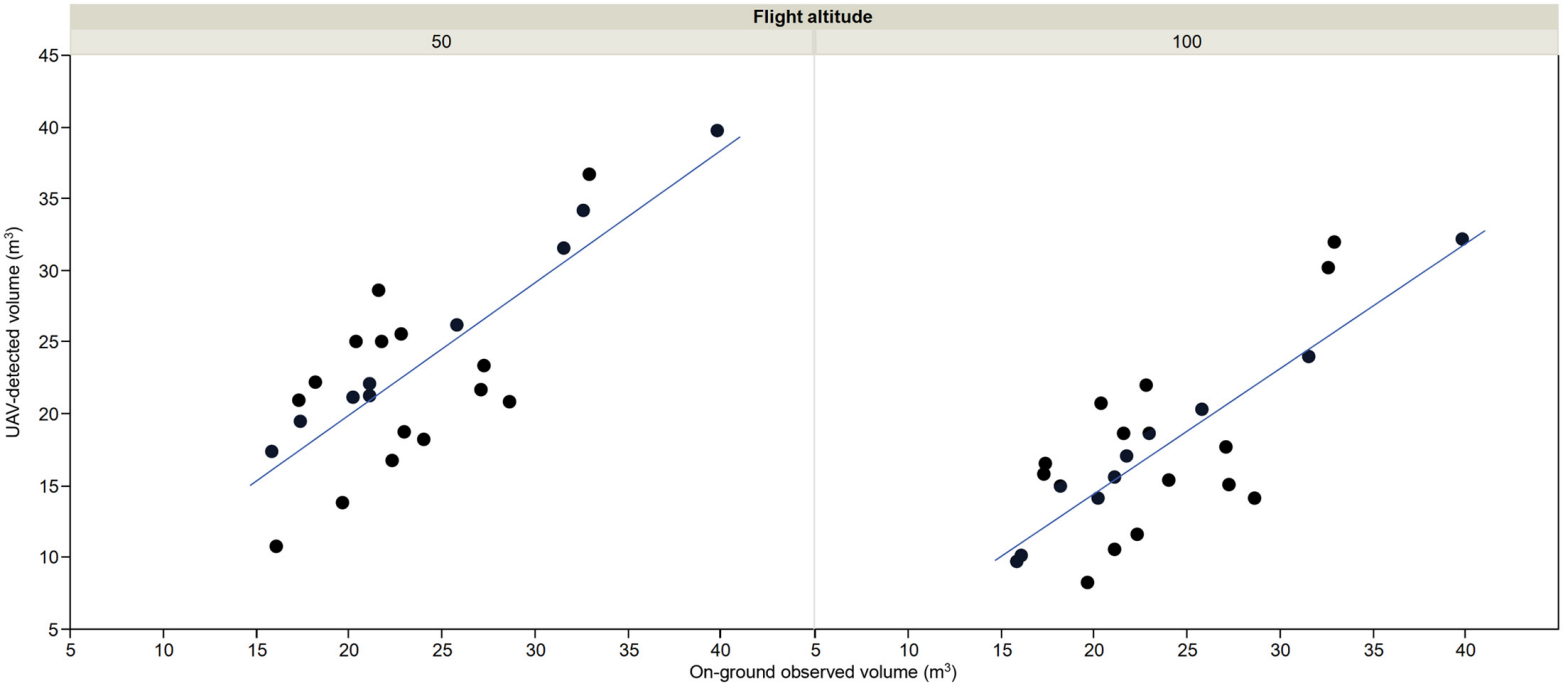
Comparison between on-ground-based volume estimations and UAV-based tree volume values computed from the UAV-images captured at 50 m (left) and 100 m (right) of flight altitude, respectively. The UAV-based values were calculated by integrating the volume of all the pixels within each image-object corresponding to each individual tree, which permitted dealing with the irregular shape of every tree and consequently avoiding the errors due to inexact comparisons of the trees to regular solids. The UAV-based values were compared to on-ground estimations, which were calculated after manually measuring tree canopy diameter and tree height with a ruler and then applying the ellipsoidal geometric model.

**Fig 10:**
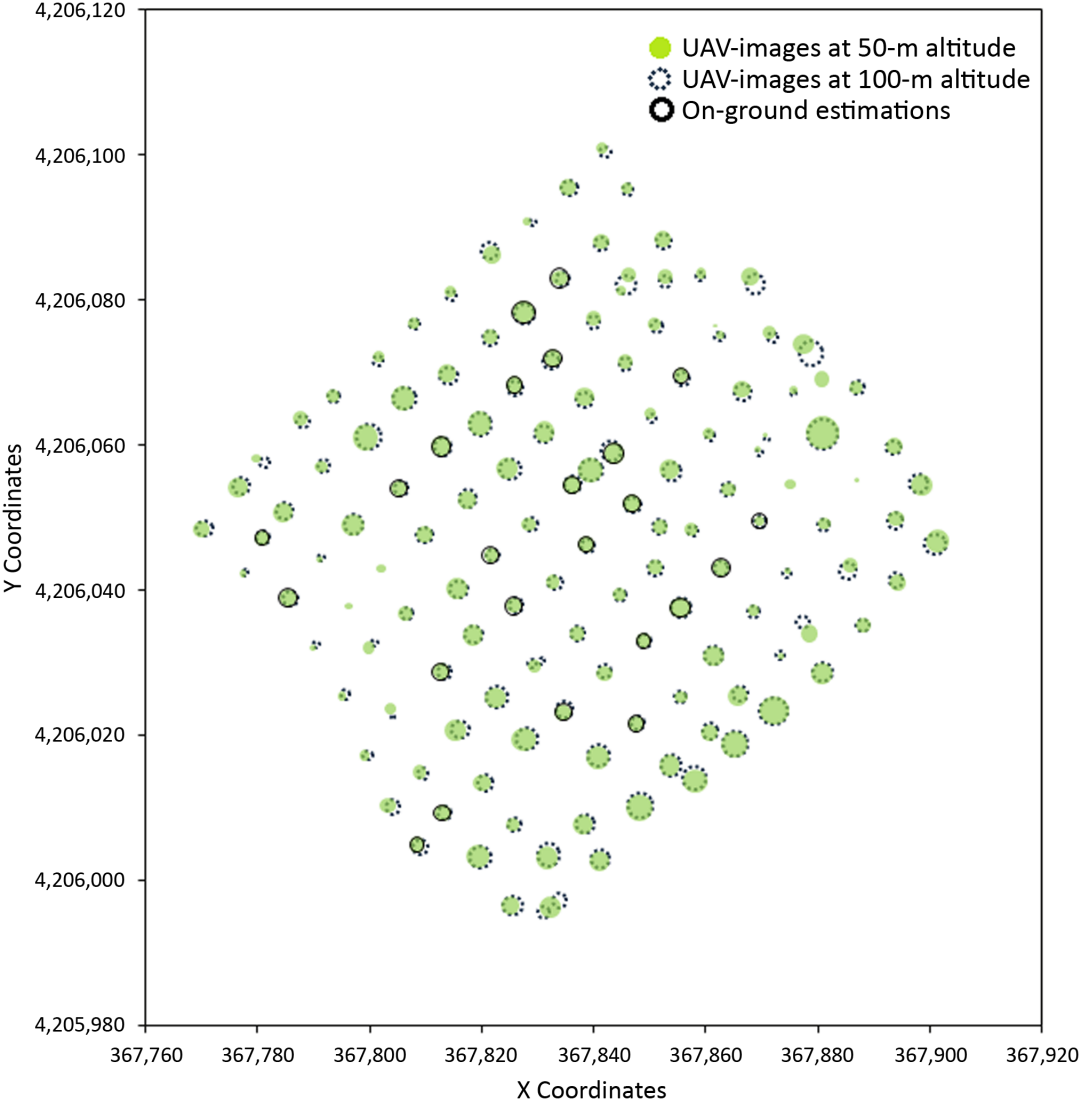
Spatial position and crown volume computed in the validation field B by using UAV-images captured at 50 m (green circles) and at 100 m (dotted circles) of flight altitude with the multispectral sensor and their relative comparison to the on-ground estimations of the validation trees (solid circles).

A precise measurement of tree projected area and tree height was crucial for modelling tree crowns and consequently for computing tree volume in the next phase. The relationship between the UAV-based and the on-ground-based volume estimations of the individual trees is shown in the Fig 9. The coefficient of determination was 0.65 and 0.63 with the 50- and the 100-m-altitude images, respectively. In this case, the differences between both variables do not denote real errors of the UAV-based measurements because the on-ground-based values were derived by applying the conventional geometric equation that considers the trees as ellipsoid forms [1], which can produce inexact on-ground estimations. On the contrary, the 3-D products derived in this investigation reconstruct the irregular shape of the tree crown, which hypothetically allows better estimations of tree volume than those ones derived from on-ground measurements. In any case, similar magnitudes were observed between both approaches with independence of the flight altitude considered; i.e., the trees that were identified as bigger on the ground were also quantified as trees with larger volumes by the UAV-based procedure and vice versa (Fig 10).

### Detailed map information provided by the OBIA algorithm

After extracting the geometric features of every individual tree or tree-row in the entire plantations, an additional advantage of the OBIA procedure was its capacity to automatically compute such information at different levels and export accurate data as vector (e.g., shapefile format) and table (e.g., excel or ASCII format) files. On the one hand, global information at the field level includes field dimensions, the number of trees, averaged tree spacing and tree statistical data (e.g., medium and standard deviation of tree heights and crown volumes). On the other hand, spatial data at the tree or tree-row level includes the central coordinates, dimensions of the main length and width axes, canopy projected area, tree height and crown volume (Table 5). This spatial information allows creating maps of each one of the geometric features studied (Fig 10), which show the heterogeneity of the whole plantation and the zones in the field with different tree growth.

**Table 5:** A sample of the output data file computed at the tree level.

Tree ID	Position^1^		Geometric features				
	X	Y	Length axis (m)	Width axis (m)	Projected area (m²)	Height (m)	Volume (m3)
1	367,769	4,206,048	4.78	4.00	13.21	3.85	21.84
2	367,774	4,206,048	5.15	4.72	12.98	1.67	11.66
3	367,777	4,206,042	2.51	1.59	2.57	3.25	5.47
…	…	…	…	…	…	…	…
135	367,784	4,206,050	4.59	4.34	12.91	3.49	33.21

^1^UTM coordinate system (zone 30N); Datum WGS84.

Accurate spatial data of each individual tree was automatically computed by the OBIA procedure in a field with 135 trees. In this case, the remote images were taken at 50 m flight altitude with a multispectral sensor.

### Time consumption

Considering the entire workflow from flight operation to features extraction, the required time to monitor one hectare of field surface varied from several minutes to a few hours depending on the sensor used and the number of the remote images collected by the UAV (Table 6). Most percentage of time was dedicated to image mosaicking and analysis, which is mainly affected by image spatial resolution. For this reason, time needed to process the visible-light images (4,032 x 3,024 pixels) was pretty longer in comparison to multispectral images (1,280 x 1,024 pixels). However, processing time was registered using a standard computer (16 GB of RAM, Intel core i5 processor and graphic card of 1 GB), so a drastic reduction of this time is possible with a more powerful computer.

**Table 6:** Averaged time per surface hectare consumed by each step of the UAV-based workflow as affected by the type of sensor and flight altitude.

			Time (h:min)/ha			
Sensor	Flight altitude (m)	# images/ha	Flight operation^1^	Image mosaicking	OBIA analysis	Total
Multispectral	50	60	0:13	0:25	0:09	0:47
	100	10	0:07	0:02	0:04	0:13
Visible-light	50	70	0:05	4:00	1:10	4:15
	100	20	0:03	0:40	0:25	1:08

^1^ With the visible-light camera, the UAV route was configured to continuously take photos with an interval of 3 seconds, flying in lines at 3 m/s with a side lap of 60%. With the multispectral camera, the UAV route was programmed to stop in every acquisition point. The multispectral images were taken with 60% side and forward overlaps.

Accordingly, an agreement between result accuracy and operation length is needed in order to select the sensor and the optimum flight configuration. In our investigation, results obtained at 50 m altitude were around 10–20% better than the ones obtained at 100 m altitude, although image processing was around four times longer at 50 m altitude. From a practical point view, the 100-m-altitude images are recommended in order to increase the ground area covered in each flight and, consequently, to reduce both the mission length and size of the image set. However, the potential precision expected from each flight altitude should also be considered according to the project quality requirements.

## Conclusions

This investigation has shown the capacity of UAV technology to efficiently produce 3-D geometrical data of hundreds of agricultural trees at the field level. In combination with an innovative object-based image analysis algorithm, we computed the canopy area, tree height and crown volume of the trees in a timely and accurate manner, which offers a very valuable alternative to hard and inefficient field work. After comparing a set of remote images collected with both a visible-light camera and a multispectral sensor, we concluded that the upper one is better recommended for fields with a tree-row plantation pattern and the latter one for single-tree plantations. We also observed minimum differences between the results obtained with the images collected at 50-m and at 100-m of flight altitude, concluding that the taller altitude should be generally selected in order to reduce the time needed to collect and to process the images.

The georeferenced information provided by our procedure allows creating maps of orchard heterogeneity and, consequently, observing zones with different tree sizes. These maps are critical to understand the linkages between tree grown and field-related factors (soil properties, weed infestations, etc.) or to study the mutual relationship between nearby trees, which can help to detect problems associated to soil or crop deficiencies or to diagnostic tree pathologies. In addition, these maps allow adopting a strategy for site-specific management of homogenous zones based on filed field or tree spatial variability in the context of precision agriculture [8], which could increase farmer net economic returns by economising on inputs (fertiliser, pesticide, water, etc) and field operations (pesticide application, irrigation, harvesting, pruning, etc).

Particularly in this context, there is a demand for developing a timely site-specific program to reduce the issues that are associated with current pest control practices in crops and to comply with the European legislation and concerns for the Sustainable Use of Pesticides (Regulation EC No 1107/2009; Directive 2009/128/EC). These regulations include such key elements as reductions in applications using an adequate amount of pesticides according to the specific requirements. Our investigation offers a reliable tool for an accurate and high-throughput monitoring of the spatial variability of agricultural-tree fields under two different plantation patterns, including tree height and crown volume of all the trees in the whole plantation, which could be used to save agricultural inputs and to optimize crop management operations with relevant agro-environmental implications.
